# Effects of the functional orthopaedic therapy on masticatory muscles activity

**DOI:** 10.4317/jced.53986

**Published:** 2017-07-01

**Authors:** Elena Di Palma, Michele Tepedino, Claudio Chimenti, Gianluca M. Tartaglia, Chiarella Sforza

**Affiliations:** 1 DDS, PhD, Department of Biotechnological and Applied Clinical Sciences, University of L’Aquila, Italy; 2 Professor, Department of Biotechnological and Applied Clinical Sciences, University of L’Aquila, Italy; 3 DDS, PhD, Department of Human Morphology, Functional Anatomy Research Center (FARC), Faculty of Medicine and Surgery, University of Milan, Italy; 4 Professor, Department of Human Morphology, Functional Anatomy Research Center (FARC), Faculty of Medicine and Surgery, University of Milan, Italy

## Abstract

**Background:**

The purpose of this study was to examine surface electromyographic (sEMG) activity of masticatory muscles before and after functional orthopaedic therapy with Sander appliance.

**Material and Methods:**

Ten adolescents (5 girls, 5 boys) with an Angle Class II, division I malocclusion, 9-13 years old, were submitted to sEMG before and after functional orthopaedic therapy. To verify the neuromuscular equilibrium, the standardized EMG activities of right and left masseter and anterior temporal muscles were recorded during maximum voluntary clench, and analysed calculating: POC (index of the symmetric distribution of the muscular activity determined by the occlusion); TC (index of presence of mandibular torque) and Ac (index suggesting the position of occlusal barycentre). The total muscular activity was also calculated. Pre- and post- functional therapy data were compared with Wilcoxon Signed-Rank Test.

**Results:**

Before treatment, all subjects had a good neuromuscular equilibrium, which was not altered by treatment.

**Conclusions:**

sEMG evaluations allow to quantify the impact of occlusion on masticatory muscle activity and to control that the functional orthopaedic therapy maintain a good muscular coordination.

** Key words:**Functional appliance, Sander appliance, electromyography, masticatory muscles.

## Introduction

The first attempt to study the muscular activity in orthodontics was reported by Moyers (1), who suggested that different patterns of muscular activity seemed to be associated with different types of occlusion. In particular, temporal muscle alterations seemed to be the etiologic factor of Class II malocclusion (1). When Class II malocclusion patients were compared with normal occlusion patients, an increased electromyographic (EMG) activity in the temporal muscle was found both in postural and in intercuspal position (1). During chewing, an irregular muscular pattern and a tendency to reduced EMG activity in the temporal and masseter muscles have also been reported in patients with Class II malocclusion (2,3). During maximal biting, Class II malocclusion pa-tients exhibited less EMG activity in the masseter and temporal muscles than normal occlusion patients (3). Alterations in the perioral muscles have also been reported (4).

The altered maxillo-mandibular anteroposterior relationship, altered and instable occlusal conditions, and a reduced mandibular displacement during muscular contraction have all been hypothesised to explain the alterations of muscular activity.

EMG was used in orthodontic diagnosis, but also to verify the effects of therapy; in particular, muscular effects in Class II malocclusion treatment by functional appliances were investigated (4-10).

The immediate response to functional treatment (with different appliances) was a strong reduction in temporal activity during biting, but a normal pattern of balance reappeared after successful treatment (2,3,10). At the end of the therapy, when the occlusion was stabilized, the contraction pattern in the analysed muscles was similar to that seen in subjects with normal occlusion (11). Recently, a positive effect of orthodontic treatment was reported also for the activity of orbicularis oris muscle during the performance of standardized movements (4).

The improvements of muscular activity previously reported were mainly focused on the electrical activity of the muscles, whereas their functional symmetry has not been analysed in detail. Indeed, this approach to the analysis of muscular activity is not complete, because an increase of muscular electrical activity, to the detriment of their symmetry, is not desiderable (12,13). Besides, electrical activity by itself shows a weak repeatability if the surface EMG (sEMG) recording is not standardized using percentage indices (8,14).

At the end of the orthopaedic-functional treatment, if the muscles were balanced during a symmetrical contraction, a long lasting stable result is more probable. Indeed, both the orthopaedic-functional treatment and the orthodontic treatment may cause an altered muscular equilibrium, and in this case the relapse will be a physiological attempt of the stomatognathic system to go back to an acceptable equilibrium (15).

The aim of the current study was to examine the effect of a Sander appliance on the symmetrical distribution of the muscular activity of masticatory muscles in a group of adolescents with a Class II malocclusion.

## Material and Methods

The experimental group consisted of 10 adolescents (5 girls, 5 boys) with Angle Class II, division 1 malocclusion. The age of the patients at the beginning of treatment ranged from 9 to 13 years (on average 10 years). The patients were selected at the orthodontic department of the University of L’Aquila (Italy).

The inclusion criteria of the study were.

- Class II molar and canine relationship;

- Overjet ≥ 5mm;

- Mixed or permanent dentition;

- Absence of tooth agenesis or supernumerary teeth;

- Absence of traumatic injuries;

- Absence of complex craniofacial deformities or syndromes;

- Absence of transversal deficit of the maxilla;

- Light crowding.

- No temporo-mandibular disorders (TMD) symptoms and signs;

- Meso- or brachifacial pattern.

For each patient, lateral head films were obtained, and a computer-assisted cephalometric analysis was carried out (OrisCeph, Elite Computer, Italy) by the same operator; several angular and linear measurements were calculated ( Table 1). Additionally, hand-wrist radiographs were used to determine the developmental stage of all patients. Only patients who were just before or just after the peak of the pubertal growth were included in the study.

**Table 1:**
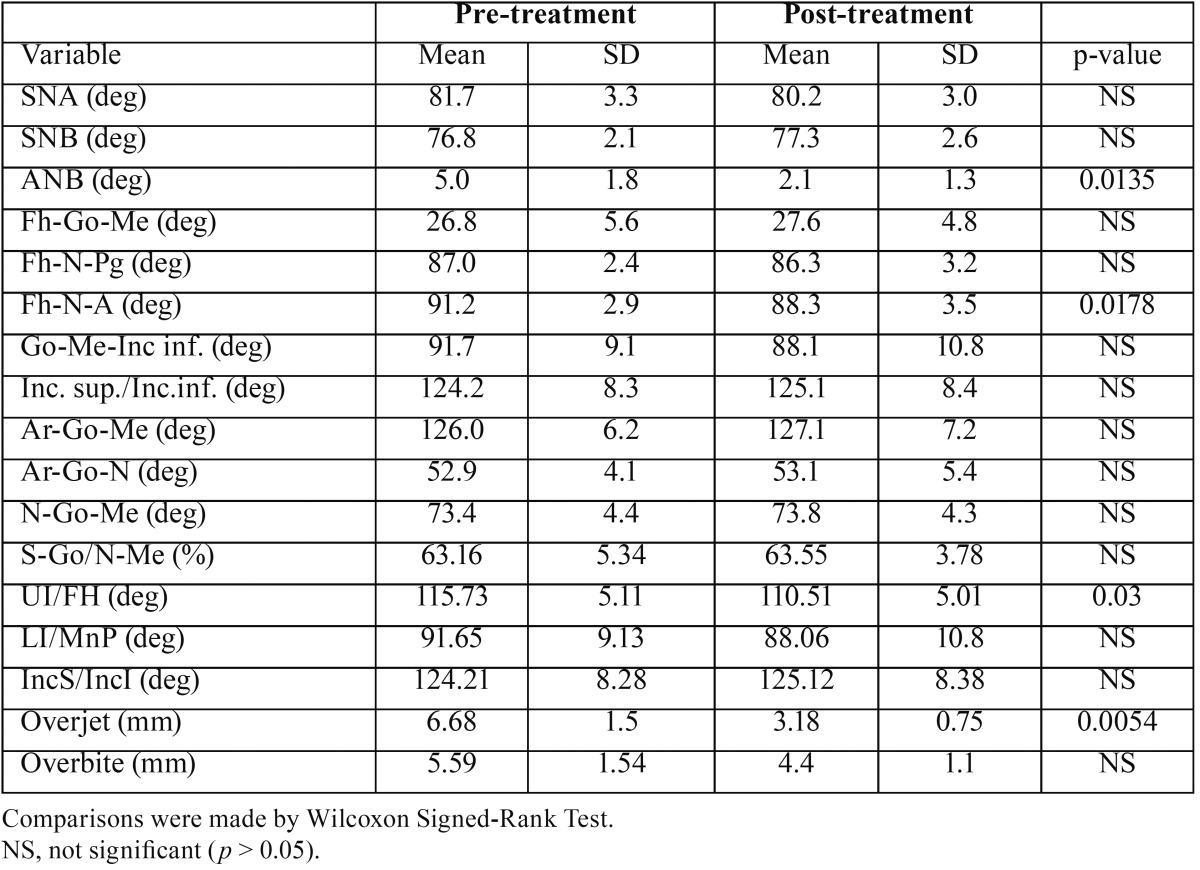
Dental and craniofacial morphology before treatment and after 1 year of treatment with Sander’s functional appliance.

**Figure 1:**
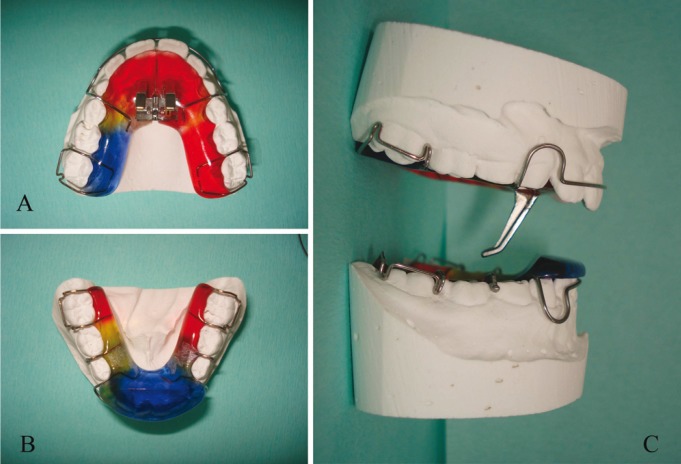
Sander’s bite jumping appliance; A, maxillary palate; B, Mandibular plate; C, lateral view showing how the two plates interconnect with each other determining a mandibular advancement.

The patients were treated with Sander’s appliance (Fig. 1), and the mandible was advanced in utmost protrusion of 6 mm with an opening of 4 mm. If necessary, the mandible was advanced until a Class III incisal relationship was obtained. When necessary, the appliance was increased step by step to extend the maxillary arch.

The patients were instructed to use the appliance at least 16 hours a day; on average, the treatment lasted 1 year.

-Sander’s appliance 

The appliance for the upper jaw was fitted out with a screw for the upper jaw and with protrusive elements long 16 mm (Fig. 1). The length of these elements is a key part of the treatment, and for a good functioning of this appliance it is necessary to maintain their full length. The superior appliance screw allows to expand the maxillary arch when necessary.

The appliance for the lower jaw showed an inclined plane. If the patient is biting these stainless-steel parts together, they are guided by the inclined plane (16).

-sEMG analysis

To evaluate the muscular equilibrium in static conditions, sEMG analysis of right and left masseter and anterior temporalis muscles was performed in all patients before and after functional therapy (14,17,18).

All patients were submitted to the EMG exam only after obtaining written informed consensus from parents/legal tutors. Oral consensus was also obtained by all children.

To reduce impedance, the skin was carefully cleaned before the electrode placement, and recordings were performed 5-6 minutes later, allowing the conductive paste to adequately moisten the skin surface. During testing disposable silver/silver chloride bipolar electrodes with a diameter of 10 mm and an interelectrode distance of 21±1 mm (Duo-Trode; Myo-Tronics Inc., Seattle, WA, USA) were used. A disposable reference electrode was applied to the forehead. The electrodes were located according to the recommendations of SENIAM (Surface EMG for Non-Invasive Assessment of muscles (19).

The EMG activity was recorded using an instrument that recorded, amplified, digitized and filtered the analogical EMG signal (14) (De Gotzen srl, Legnano, Milano, Italy).

-Two EMG recordings were made in each session (14):

- standardization recording: 5-s maximum voluntary clench (MVC) performed on two 10-mm thick cotton rolls positioned between the mandibular posterior teeth;

- experimental recording: 5-s MVC performed in maximum occlusion without cotton rolls.

The 3 s with the most stable EMG signal were automatically selected by the software.

For each patient, the EMG potentials of the analyzed muscles recorded during the MVC tests were expressed as percent of the mean potential recorded during the standardization test (MVC on the cotton rolls), unit: μV/μV x 100. All subsequent calculations were made with the standardized potentials. Relative percentage EMG values should be affected only by the occlusal surfaces, because this kind of standardization should annul the variability caused by skin and electrode impedance, electrode positioning (17).

-Data analysis: sEMG indices 

For each patient, several EMG indexes (symmetry, torque, relative activity) were computed. Besides, the total standardised muscle electric activity developed by the four investigated muscles during the MVC was obtained.

To assess muscle symmetry, within each subject the EMG waves of paired muscles were compared by computing a percentage overlapping coefficient (POC, unit: %). POC is an index of the symmetric distribution of muscular activity as determined by occlusion. It ranges between 0 (no symmetry) and 100% (perfect symmetry); values higher than 85% are considered normal. POC index was calculated for each couple of homologous muscles (masseter POC, temporalis POC).

The activity index (Ac) was calculated to compare the muscular activities of masseter and temporalis muscles; the index informs about the prevailing area of the contacts, and thus about the principal occlusal centre of pressure. When the standardized EMG potentials are not balanced between the two analyzed masticatory muscles, the occlusal center of pressure (clench on the occlusal surfaces as compared to clench on the cotton rolls) might be displaced onwards (temporalis prevalent) or backwards (masseter prevalent). Ac ranges from -100% (temporalis muscle prevalence) to +100 (masseter muscle prevalence) (20). A negative activity coefficient has already been reported to be determined from dental contacts in the anterior arch, with a larger load on the temporomandibular joint (18).

Because an unbalanced contractile activity of contralateral masseter and temporalis muscles, i.e. right temporalis and left masseter, might give rise to a potential lateral displacing component, the Torque Coefficient (TC) was computed (14). TC ranges between 0% (unbalanced standardized masseter and temporalis potentials) and 100% (well comparable standardized masseter and temporalis potentials). When the index is lower than 90%, there is a muscular latero-deviant couple which may push the mandible towards left or right.

The total standardised electric activity developed by the four investigated muscles during the MVC was computed as the average integrated areas of the masseter and temporalis EMG potentials over time (unit: µV/µV s %). The value developed during the MVC on occlusal surface was obtained as a percentage of the muscular activity developed during the cotton roll clenching (14).

The repeatability of sEMG recordings of masseter and anterior temporalis muscles was tested in FARC laboratory and in Orthodontic Department of University of L’Aquila (14,21). For all EMG variables, the intraclass correlation coefficients were larger than 0.62, showing a good accuracy of the measurements, without random errors (paired Student’s t test, *p* > 0.05).

Pre- (T0) and post- (T1) expansion data were compared by Wilcoxon Signed-Rank Test with a predefined significance level of 5% (*p*<0.05).

## Results and Discussion

All patients had a good compliance to functional orthopaedic treatment, and the recovery of Class I molar and canine relationships was observed in all occasions. Besides, a reduction of the over-jet and an improvement of the soft and hard tissue profile were found. The mean changes in dental and skeletal facial morphology after 1 year of treatment are listed in  Table 1.

Before treatment, all children had a good muscular equilibrium, with EMG indices within reference values (14,18). After treatment, all EMG indices remained within reference values, and no significant pre-post treatment differences were found ( Table 2).

**Table 2:**
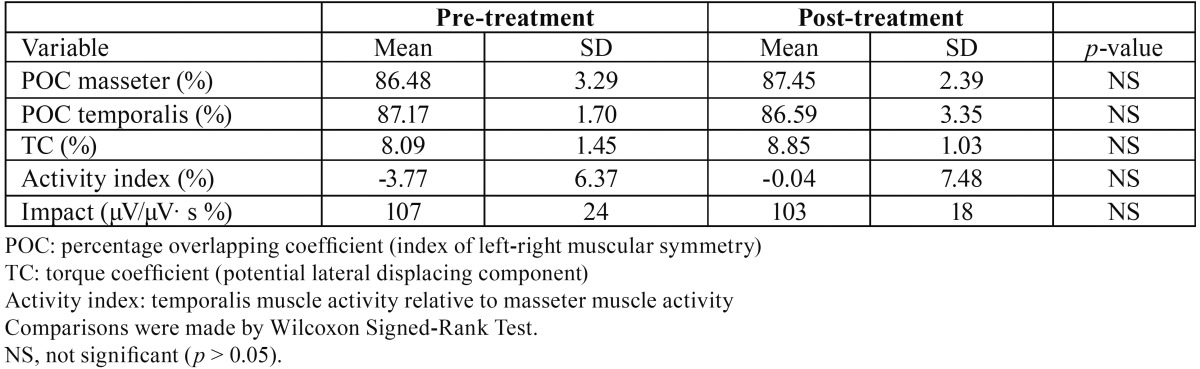
Electromyographic variables calculated for 10 patients before and after functional orthopaedic therapy.

The present study assessed a group of adolescent patients with Angle Class II malocclusion; the study was designed to gain some information on the neuromuscular effects of the treatment with Sander appliance.

Currently, EMG allows to supervise and investigate some of the main muscles involved in chewing, deglutition and head posture and motion (masseter, temporalis, anterior digastric and sternocleidomastoid), obtaining well reproducible results when standardised protocols are used (14,18). Additionally, sEMG is being used to assess the activity of facial muscles, analyzing the effects of malocclusion and orthodontic treatment on muscular activity (4,22).

In the current study, the comparison between the EMG pre and post-treatment data allowed to evaluate the effects of the orthopaedic-functional therapy on muscular function. After treatment, all subjects maintained a good muscular equilibrium (POC index: right-left side within muscle; Activity index: masseter vs. temporalis; Torque coefficient: laterodeviant couples), without statistical significant variations (2,3,7,10,11,23-25).

Together with conventional clinical and cephalometric assessments, the evaluation of the effects of orthopaedic-functional therapy should include objective functional measurements to quantify the final result of the new occlusal condition in the wider context of the stomatognathic apparatus of the patient. In the clinical practice, surface EMG can be used to this scope (15).

Indeed, occlusal stability has been shown to be related to muscular performance: subjects with a high occlusal stability reveal shorter times of contraction and wider EMG potentials during mastication when compared to patients with a lower occlusal stability (14,26,27). In 9-year-old children with Class II malocclusion, interceptive orthodontic treatment improved the form and function of perioral muscles (4).

One of the limitation of the current study, as of other investigations (4-6,8,9,15,24) is the lack of a long-term follow up: patients were analyzed only up to 1 year after the end of therapy. Considering its lack of dangerous and painful procedures, sEMG can be longitudinally repeated in post-treatment patients. The patients analyzed in the present study are currently longitudinally followed up to control their masticatory and muscular function until treatment stabilization and maturation of the stomatognathic system.

Additionally, the current study assessed a limited group of patients with both mixed and permanent dentitions. Indeed, each subject acted as control of her/ himself, and this limited the effect of sample heterogeneity. Long-term studies with larger samples are required.

## Conclusions

The current results showed a favourable muscular response to mandibular advancement with Sander appliance in a group of adolescents with a Class II malocclusion.

It is expected that a good muscular equilibrium between the masticatory and facial muscles guarantees a long lasting stable result; sEMG could be efficaciously used to control the functional stability of orthodontic patients during their follow-up with a simple, not-invasive and low-cost assessment.

Within the limitations of the current study, the functional orthopaedic treatment represents, therefore, an efficient therapeutic method not only from the dental and skeletal points of view, but also from the functional side, because the masticatory muscles can adapt well into the new occlusal condition, at least in children with a good muscular coordination before treatment.

Additionally, surface EMG was found to allow a simple and non invasive evaluation of masticatory muscle activity. EMG evaluations allowed to quantify the impact of occlusion on masticatory muscle activity, and to control that the functional orthopaedic therapy maintained a good function.

Future studies should examine the stability of the treatment and the longitudinal changes in activities of these muscles.
